# Alcohol, Brain Derived Neurotrophic Factor and Obesity among People Living with HIV

**DOI:** 10.4172/2155-6113.1000245

**Published:** 2013-09-20

**Authors:** María José Míguez-Burbano, Luis Espinoza, Robert L. Cook, Mayra Mayra, Diego Bueno, John E. Lewis, Deshratan Asthana

**Affiliations:** 1 School of Integrated Science and Humanity, Florida International University, Miami, FL, USA; 2 Department of Medicine, University of Miami School of Medicine, Miami, FL, USA; 3 Departments of Epidemiology and Medicine, University of Florida, Gainesville, FL, USA; 4 Department of Psychiatry & Behavioral Sciences, University of Miami School of Medicine, Miami, FL, USA

## Abstract

**Introduction:**

In an expanding HAART era, obesity has become a health problem among persons living with HIV (PLWH). Whereas the rising level of obesity has been largely attributed to poor nutrition and exercise habits, differences in biological factors may explain why some individuals gain more weight than others. Thus, our main goal is to prospectively determine in PLWH whether plasma brain-derived neurotrophic factor (BDNF), and hazardous alcohol use (HAU), two overlooked but highly prevalent conditions among PLWH, correlate with an adverse anthropometric profile. Also to test whether these relationships varied in men and women

**Methods:**

The Platelets mediating Alcohol and HIV Damage Study (PADS) is an ongoing multiethnic study of 400 PLWH receiving regular medical care in South Florida (37% females and 63% males). Semi-annual visits consisted of a medical exam, including anthropometrics to assess both general (body mass index: BMI) and central obesity (waist and hip circumferences). Participants also completed health history questionnaires, and provided a fasting blood sample to obtain BDNF and immune and biochemical assessments.

**Results:**

A sizable proportion of participants met the National Institutes of Health definition of overweight (BMI = 25–29.9 kg/m2; 26%) and obese (BMI ≥ 30 kg/m2; 35%). Women were more likely to be obese than men (OR=4.9, 95% CI=2.9–8.2, p=0.0001). Compared to men, women also exhibited the highest mean plasma BDNF levels (9,959 ± 6,578 vs. 7,470 ± 6,068 pg/ml, p=0.0001). Additional analyses indicated that HAU, particularly heavy drinkers, had the smallest waist and hip circumferences if they were males, but the opposite if they were females. High BDNF levels were positively correlated with BMI. Linear regression analysis revealed that gender, BDNF, and HAU were the best predictors of BMI.

**Conclusion:**

In summary, our findings offer novel insights into the relationships between BDNF, and alcohol use among overweight and obese PLWH. Our results also suggest that these relationships may be inherently different by gender.

## Introduction

A major milestone in the HIV/AIDS epidemic was the development of highly-active antiretroviral therapy (HAART). With this therapy, scientists and health care providers no longer had to be concerned about AIDS wasting syndrome. Rather, one of the current critical issues is that a sizable proportion of persons living with HIV (PLWH) are overweight or obese [1]. This is of concern given that the overall risk of many chronic diseases rises substantially with overweight and obesity [1–6]. Particularly, abdominal fat accumulation has been associated with greater risks of developing insulin resistance, atherosclerosis, and cardiovascular diseases CVD [7,8]. Research shows that people with more weight accumulated around the waist region (the “apple shape”) face more health risks than those who carry more weight around the hips (the “pear-shape”) [9]. Mortality rates also increase with higher body mass index (BMI), and it has been estimated that at least half of all deaths (53%) among obese individuals could had been prevented with weight reduction [10].

In people with and without HIV, environmental influences, eating behaviors, energy metabolism, personality, and mood, can act as risk factors for suboptimal body weight [11–17]. Alcohol use, quite prevalent among PLWH, is another factor that has the potential to modulate weight [18–21]. Available epidemiologic studies among the general population have provided contradictory results, showing positive, negative and non-significant associations between alcohol intake and weight gain [22,23]. To further complicate this issue, such information is lacking among PLWH [24]. Thus, this dearth of data limits the ability to develop new interventions or clinical guideline recommendations. Experts have also highlighted the need to determine gender and racial differences that might exist.

Whereas the rising level of obesity in recent decades is attributable to lifestyle and environmental changes, differences in biological factors, may also explain why some individuals gain more weight than others in similar obesogenic settings. Several lines of evidence indicate that brain-derived neurotrophic factor (BDNF), and its TrkB receptor, exert control over multiple regulatory elements that maintain weight [25]. In addition to being abundantly expressed in brain regions highly relevant for the maintenance of normal body weight, animal studies have demonstrated BDNF’s critical influence on food intake, metabolic balance, and mood [26]. Gene manipulation studies have shown that BDNF’s critical role in body weight control goes far beyond a simple association. Animals with heterozygous knockout of BDNF [27], haploinsufficiency, or postnatal BDNF deletion in the central nervous system [28] exhibit progressive obesity. Increasing BDNF, with either peripheral injection or infusion into the central nervous system, causes marked alterations in food intake and body weight [29]. BDNF can also impact weight by altering hedonic aspects of eating [30].

In humans, establishing the relationship between BDNF and weight homeostasis has been more difficult. While BDNF haploinsufficiency has been associated not only with obesity but also with eating disorders, the few studies that have examined concentrations of BDNF have reached inconclusive results [31]. One study showed a significant positive relationship between BDNF and obesity [32], and another found that BDNF negatively correlated with both BMI and body fat [33]. BDNF has been found to be elevated in obese women, but dropped after bariatric surgery [34]. Conversely, Mercader et al. [35] observed no correlation between BDNF and BMI in eating disorder patients.

Taken together, these findings demonstrate the need for additional studies to establish the role of BDNF (with or without alcohol use) in overweight and normal-weight adults. Further spurring our interest was evidence from animal models indicating that alcohol has important effects on BDNF [36]. However, BDNF has only been measured in hazardous alcohol users in a handful of studies, and none of them looked at PLWH. The Platelets mediating Alcohol and HIV Damage Study (PADS) is a large, single-site multi-ethnic cohort with a high enrollment of women, providing a unique opportunity to assess the roles of BDNF and alcohol among PLWH.

## Methods

This paper represents a cross-sectional analysis of baseline data from PADS. The PADS is a cohort consisting of 400 PLWH, who are at least 18 years old and under regular care at Miami’s primary open-access public health system. Our choice of PLWH in an open-access public health system with standard treatment protocols was purposefully designed to minimize social, medical, and treatment inequalities. Since the main focus of this cohort study was to assess the potential effects of alcohol, participants were enrolled regardless of alcohol consumption, and alcohol groups were demographically matched. To reduce the confounding effects of illicit drug use, the DSM-IV-TR questionnaire was applied, and those participants who were dependent on drugs or injecting illicit psychoactive substances were excluded and confirmed with negative urine toxicological screens at admission.

Non-ambulatory patients and those presenting with major medical co-morbidities were excluded. Participants were questioned about several medical disorders, and research study staff reviewed medical records to confirm their eligibility. If participants had a history of central nervous system (CNS) opportunistic infection, head injury with or without loss of consciousness, tumor, major psychiatric disease, a developmental disorders, severe malnutrition, chronic renal failure, or thyroid, cardiovascular, or immune-based diseases were excluded. In addition, participants who had cirrhosis, active viral hepatitis, or liver enzymes two standard deviations above normal values were ineligible. The protocol was approved by the Institutional Review Boards at Florida International University and the University of Miami. The study was conducted according to the principles expressed in the Declaration of Helsinki. Those participants who provided written informed consent and signed a medical release form were consecutively enrolled and followed over a period of six months.

### Participants’ assessment protocol

Upon entry into the study, we obtained sociodemographic, alcohol use, and medical history information (e.g., HAART details) using computerized structured questionnaires. Blood was drawn in fasting subjects in order to best evaluate immunological, metabolic, and nutritional profiles.

### Anthropometrics and physical activity levels

Basic body composition was measured by trained staff using quality assurance methods that were tightly defined to reduce systematic errors. Body weight in kilograms was measured after an overnight fast, and height was measured to the nearest centimeter. Measured weight and height were used to calculate BMI (weight (kg)/height (m)2). Participants were classified as thin if BMI was <18.5 kg/m2, eutrophic if BMI was 18.5–24.9 kg/m2, overweight if BMI was 25–29.9 kg/m2, and obese if BMI was ≥30 kg/m2. Since BMI is a measure of overall body composition, but does not account for the wide variation in body fat distribution, we evaluated waist/hip ratio and waist circumference as indicators of visceral adiposity. Waist and hip circumferences were measured using a tape positioned at the high point of the iliac crest for the waist and at the greatest circumference of the buttocks. Each measurement was made with minimal respiration to the nearest 0.1 cm with the tape snug, but not compressing the skin. The National Institute of Diabetes, Digestive and Kidney Diseases states that a woman with a waist/hip ratio >0.8 and a man >1.0 are obese and at increased risk of chronic disease due to suboptimal fat distribution [37].

### Alcohol and tobacco use

At each visit, PLWH reported alcohol intake in the past six months using two standardized and validated brief screening questionnaires: the Alcohol Use Disorders Identification Test (AUDIT) and the Alcohol Dependence Scale (ADS) [38,39]. The AUDIT includes three questions on alcohol consumption, three on drinking behavior and dependence, and four on the consequences or problems related to drinking. The ADS assesses alcohol withdrawal symptoms, impaired control over drinking, awareness of a compulsion to drink, increased tolerance to alcohol, and salience of drink-seeking behavior.

Participants were asked to report a serving size using models of 12 ounces of beer, 5 ounces of wine, and 1.5 ounces of liquor. Alcohol consumption scores were computed by averaging cross products of quantity and frequency of beer/wine and hard liquor reported on the AUDIT and ADS responses. Then, based on the National Institute of Alcohol Abuse and Alcoholism criteria, men who reported >14 drinks/week or >4 drinks in one day and women who reported >7 drinks/week or >3 drinks in one day were classified as hazardous alcohol use (HAU), while those who reported fewer drinks were categorized as non-HAU. Participants who drank more than five standard drinks in a given day were considered binge drinkers [40].

The Fagerstrom Test for Nicotine Dependence [41] was used to determine the number of cigarettes smoked per day, the age of initiation, number of years smoking, the time between awakening and smoking the first cigarette of the day, and the episodes in which the smoker lost control of smoking behavior (such as smoking at inappropriate times or places).

Following National Health Interview Survey guidelines, a participant was defined as a current smoker if he/she had smoked at least 100 cigarettes in his/her lifetime and now smoke either every day or some days. A non-smoker was defined as anyone who has never smoked or at some time smoked cigarettes, cigars, or pipes for less than 3 months. Based on these definitions, participants were assigned into four groups based on their HIV serostatus (positive/negative) and their smoking status (smoker/non-smoker).

### BDNF

Prior studies have demonstrated that plasma levels of BDNF, although different from levels in cerebrospinal fluid (CSF), are correlated with CSF in CNS diseases [42]. Due to this association, circulating levels of BDNF were selected for analyses. Plasma BDNF levels were measured using a commercially available ELISA kit (R&D System) according to the manufacturer’s instructions. Briefly, 50 µl of standard and 20-fold diluted samples were pipetted into wells of a 96-well immunoplates. An enzyme-linked monoclonal antibody specific for BDNF was added to the wells. Following a wash to remove any unbound antibody-enzyme reagent, a substrate solution was added to the wells and color developed in proportion to the amount of BDNF bound in the initial step. The color development was stopped and the intensity of the color was measured. BDNF concentration in plasma was calculated based on a standard curve. The minimum detectable dose of BDNF is typically less than 20 pg/mL.

### Viroimmune variables

The main study outcomes related to HIV and HAART response were classified into the following categories: number of years since first diagnosis, viral load, CD4, and TNF-α. HIV viral burden was quantified using the Amplicor HIV monitor test (Roche Diagnostic System). The lower threshold for detection at the time of the study was 400copies/ml. The percentage and absolute numbers of T CD3+/CD4+, lymphocyte cell counts and percentages were determined by flow cytometry per National Institute of Allergy and Infectious Diseases laboratory protocols.

The date of initiation of HAART, antiretroviral medications used, and prior exposure to HAART were all recorded.

### Statistical analyses

The data were analyzed using SPSS 18.0 for Windows (IBM, Inc., Chicago, IL, USA) and p values <0.05 were considered statistically significant. Although this is a prospective cohort study, we used only baseline data for these analyses. The distributions of our primary outcomes of interest were examined with normal probability plots. Descriptive statistics, such as minimum, maximum, median, and mean with SD, were used to summarize the data and to detect outliers and missing values. Log transformations were performed with variables not normally distributed. Percentages and frequencies were used to describe categorical variables. Please note that for gender analyses, ten transgender participants were excluded.

Viral load results (RNA copies per mL) were log-transformed before analysis. Sociodemographic and clinical variables (e.g., age, race/ethnicity, gender, and adherence) were evaluated for possible inclusion in the univariate analyses and in the multivariate model as covariates. T-tests and chi squares were used to compare means and proportions respectively between the alcohol groups (HAU vs. non-HAU, heavy vs. non-heavy alcohol use). Differences in demographic, clinical history, and body composition were assessed using chi squares for categorical variables, two sample Student's t-test for normally distributed continuous variables, and the Wilcoxon rank sum test for non-parametrically distributed continuous variables. We employed the Bonferroni correction to reduce the risk of type I errors because of the multiple comparisons.

Linear regression models were employed to predict body composition and analyses were controlled by alcohol consumption level, age, years of HIV diagnosis, and antiretroviral treatment (yes/no). The validity of model assumptions was evaluated with the residuals.

## Results

### Sample characteristics

Among the 400 PLWH participants, 37% were female and 63% were male ranging in age from 21 to 50 years (42 ± 6 years). Participants with complete information were included in this study, however since the study focus on gender analyses, ten transgender participants were excluded. Most participants were African-Americans (67%) or Hispanics (25%) with a smaller proportion of Caucasians (4%) and Caribbeans (4%). Overall nutritional status, determined by level of serum albumin, was within the normal range for 99% of the participants (4.3 ± 0.4 g/dL); malnutrition (serum albumin <3.5 g/dL) was observed in only 1% of the group. The cohort was characterized by normal liver enzymes levels suggestive of preserved liver function. Drugs of abuse, particularly marijuana and crack, were reported by only one-third of the sample and mostly as recreational use over the weekends.

#### Anthropometrics and sociodemographic characteristics

Only one-third of the study sample had a normal BMI value (18.5–24.9 kg/m2). Many participants met the definitions of overweight (26%; 25–29.9 kg/m2) and obese (35%; ≥30 kg/m2). Overweight and obesity were more prevalent than wasting (2%, p<0.0005). Table 1 shows demographic and clinical characteristics of our sample based on BMI categories. Rates of obesity and overweight varied by gender and race.

#### Gender analyses

Although men were taller than women (68.2 ± 5.3 vs. 64.4 ± 3.1 inches, p=0.0001), women had higher body weight (191.9 ± 51 vs.182.2 ± 43), and mean body mass index (BMI 31.8 ± 8.6 vs. 26.8 ± 6, p=0.0001, kg/m2). Additional analyses indicated that women were more likely to be obese than men (OR=4.9, 95% CI: 2.9–8.2, p=0.0001). We also observed a trend for overweight among women (OR=1.6 95% CI: 0.9–2.9, p=0.05).

#### HIV characteristics

Despite similar rates of antiretroviral treatment (Truvada:44%, Atripla: 22%, alone or in combination with Norvir: 32% or Kaletra:13%) and adherence (high during the week: 93%, weekend:83%) both overweight and obese individuals exhibited higher T CD4 cell counts and lower viral load logarithms (Table 1). Longer cumulative time on HAART was not associated with either being overweight or obese. Differences were not observed either when considering cumulative time on individual drug classes.

#### BDNF and anthropometrics

**Figure 1:**
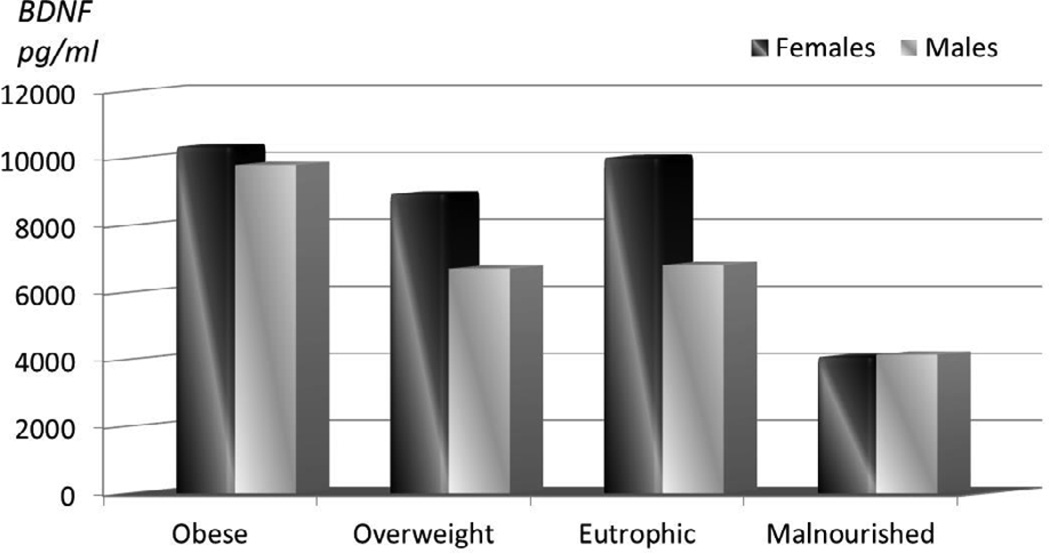
BDNF levels by Gender and BMI.

There was a wide concentration range of BDNF in circulation from 298 to >20,000 (mean 8384 ± 6366 pg/ml). BDNF levels differed by gender with women exhibiting the highest levels (9958.9 ± 6578 vs. 7470 ± 6068 pg/ml, p=0.0001). Correlations showed significant relationships between BDNF and anthropometrical variables, such as BMI (r=0.19, p=0.001) and waist (r=0.11, p=0.03) and hip (r=0.17, p=0.001) circumference. As expected, BDNF was also significantly associated with platelet count (r=0.2, p<0.001). However, BDNF was significantly higher among obese participants compared to normal weight subjects (10,099 ± 6641 vs. 7,621.9 ± 6197 pg/ml, p=0.002). Based on evidence of heterogeneity, we examined the relationship of BDNF and obesity traits by gender. As depicted in Figure 1, not only BDNF levels varied by body weight group, being higher in the obese subjects but also in the overweight individuals, they varied by gender. To further characterize the risk of BDNF status on obesity, we dichotomized BDNF levels above and below 8,000 and estimated the likelihood of being overweight, obese, or abdominally obese using logistic regression models. Subjects with BDNF levels above 8000 pg/ml had an increased risk of obesity (OR 1.4 95% CI 1.1–1.8, p=0.001). Notably, none of the malnourished subjects had levels above 8000 pg/ml.

#### Alcohol use, body composition, and smoking

Table 2 summarizes the differences in body composition measurements between HAU and non-HAU and other drinking characteristics. We found that, in the entire sample, HAU had smaller waist and hip circumferences compared to non-HAU. Moreover, a dose relationship seems to exist, since heavy drinkers exhibited even smaller measurements. However, this advantage was gender specific because among women we observed the opposite effect. While male drinkers and non-drinkers had similar abdominal circumference, all other anthropometric measurements were significantly lower. Female HAU were more likely to have larger waist and hip, but particularly significantly large abdominal circumferences (Table 3).

Smokers tended to have the lowest BMI compared to past users or never smokers. Obese participants were twice less likely to be a smoker (OR=1.8, 95% CI: 1.1–3, p=0.009). Given our findings, we repeated the prior alcohol analyses after excluding the smokers, but the results were similar (data not shown).

#### Multivariate analyses

Factors associated with BMI in the final multivariate model included a high BDNF level (β=0.46, t=2.7, p=0.007), being female (β=0.30, t=6.1, p<0.001), and HAU (β=0.23, t=2.9, p=0.005). We found no significant effects for age, race, or stage of disease.

## Discussion

Our study is unique in assessing the role of BDNF over a wide range of body composition characteristics in PLWH of both genders. First, our data showed that being overweight or obese is common (61% of our sample) in an urban HIV population in South Florida, indicating that we should redirect our concerns to assure PLWH are maintaining a healthy, normal weight. Second, our analyses revealed positive associations between BDNF and obesity, suggesting that BDNF is a candidate molecule involved in the pathophysiology of weight disorders among PLWH. Notably, this study demonstrated gender-specific associations between hazardous alcohol use and obesity traits. Men hazardous alcohol users had lower BMI, waist, hip, and weight than non-HAU, whereas women hazardous alcohol users had higher BMI than non-HAU and men counterparts. Particularly, female hazardous alcohol users exhibited large abdominal circumferences indicative of central obesity. These findings are of great concern because PLWH receiving HAART have increased cardiovascular disease risks that can be further exacerbated by being overweight [43,44]. In our opinion, these findings are also relevant, given the accumulating evidence that indicates that alcohol use triggers alterations of BDNF [45,46]. Our data are perhaps relevant from a public health perspective, since our study shows two factors that can be manipulated by interventions.

Blood BDNF levels of normal weight persons have been compared with those of obese persons, and the results of our study are consistent with the documented positive association between BDNF and obesity [47,48]. Although our cross-sectional design does not allow us to make causal inferences, prior data using animal models provide strong support that the observed relationship is more than a simple association [27–29]. It is possible that BDNF increases in obesity to compensate for its associated pathophysiologic conditions because of its potential role in improving energy metabolism [49]. Notably, while at an early age BDNF+/− mice have normal forebrain 5-HT levels and fiber density they undergo premature age-associated decrements [50]. A tempting hypothesis for this increase in BDNF is that it is a compensatory mechanism to protect the brain from further damage or to repair damage that has been done.

Although we posit that a link between HAU and obesity exist because excess caloric intake easily occur with consumption of alcohol, our hypothesis was only confirmed among the female population. Among males, the drinking frequency was inversely related to obesity. These findings highlights the need of gender analyses; first, because the relationships between alcohol consumption and body morphology appeared to be different in men and women and second given the significant differences in BDNF level between males and females. The increase central fat accumulation among females is of concern when considering that alcohol is deposited in fatty tissues and thus, alcohol might remain in the body for a longer period of time increasing the risks of alcohol’s deleterious effects. It is also possible that women who have frequent mood disorders may be using food as a method for coping with stress. It could be a proxy for some other, variable that affects body composition. Indeed data analyses revealed a relationship between lower risk of obesity and smoking, which is well known for reducing appetite.

### Limitations

The principal limitation of this study was the cross-sectional design, which does not allow us to make causal inferences between BDNF and risks of obesity. We also have a limited number of dietary intakes available for analyses at this point. The study sample was limited to the clinic settings of South Florida. Nonetheless, our study benefitted from its large sample size and from the inclusion of HAU and non-HAU PLWH from the same population source. It is also notable that we had a large number of women in the study and we directly measured height and weight and other anthropomorphic characteristics.

In summary, our study demonstrated a significant association between BDNF levels and obesity traits in a sex-specific manner. Alcohol seems to be impacting BDNF and influence obesity among people living with HIV. These results may open new pathways to address obesity among PLWH through tailored and target interventions.

## Figures and Tables

**Table 1:** Sociodemographic and Clinical Characteristics of HIV Infected Patients by BMI

Variable	Obese (n=141)	Overweight (n=108)	Normal (n=148)	Underweight (n=9)	P value

Age	42.6 ± 6.4	42.3 ± 7.4	42.7 ± 6	40.6 ± 5.2	0.7

Men	44%	68%	79%	62%	0.001
Women	56%	32%	21%	38%	

African American	74%	57%	71%	37%	0.006
Black Caribbean	2%	2%	4%	13%	
Hispanic	17%	36%	21%	25%	
White	7%	5%	4%	25%	

Annual Income:					
Less than $10,000	85%	88%	90%	100%	0.7
$11,000–$20,000	11%	8%	6%	0%	
$20,000–$49,000	2%	2%	4%	0%	
>$50,000	2%	2%	0%	0%	

Education (years of school)	11.5 ± 2	11.3 ± 2.4	11.3 ± 2.6	9.7 ± 2.1	0.1

Albumin	4.5 ± 0.5	4.3 ± 0.4	4.2 ± 0.5	4.1 ± 0.6	0.1

AST	32.5 ± 23	34.1 ± 18	34.6 ± 16	33.3 ± 9.8	0.6

ALT	37.1 ± 33	37.8 ± 20	39.2 ± 19.9	24.7 ± 6.8	0.8

CD4 cell counts	536 ± 309	406.6 ± 270	383.9 ± 251	222 ± 217	0.001

Viral Load Log	2.5 ± 1.3	2.6 ± 1.3	2.8 ± 1.2	3.8 ± 1.3	0.04

*Note:* Demographic characteristics were expressed as percentages by BMI group. Biological measures were presented as means and standard deviations.

*Note:* AST = Aspartate transaminase also called glutamic oxaloacetic transaminase (SGOT) and ALT= alanine aminotransferase also called glutamic pyruvic transaminase (SGPT).

Please note that there were 10 transgender participants that were not included in the table because gender and race could not be accurately classified.

**Table 2:** Alcohol Use and Body Composition

Variable		HAU			Total Drinks/Week	
	Yes	No	P value	>= 30	< 30	P value
BMI	27.6 ± 7.0	29.3 ± 7.9	0.03	25.8 ± 6.2	28.9 ± 7.6	0.003
Weight	182.2 ± 42.7	189.9 ± 51.6	0.1	173.9 ± 37.3	187.9 ± 48.6	0.01
Waist Circumference	37.6 ± 5.7	38.8 ± 7.4	0.09	36.4 ± 5.3	38.5 ± 6.8	0.007
Hip Circumference	40.3 ± 6.1	41.9 ± 7.0	0.02	39.3 ± 5.9	41.4 ± 6.6	0.01
Abdomen Circumference	33.4 ± 15.7	33.1 ± 16.1	0.8	30.1 ± 16	33.8 ± 15.8	0.09

**Table 3:** Body Composition according to Gender and Alcohol Use.

Variable	Female HAU >=7 drinks/week	Female Non-HAU <7 drinks/week	P value	Male HAU >=14 drinks/ week	Male non-HAU <14 drinks/week	P value
BMI	32.2 ± 8.0	31.7 ± 9.0	0.8	26.1 ± 5.0	27.8 ± 7.0	0.01
Waist Circumference	41.7 ± 7.0	39.9 ± 7.0	0.3	36.4 ± 5.0	37.9 ± 6.9	0.05
Hip Circumference	45.9 ± 4.5	44.1 ± 7.1	0.7	38.2 ± 4.9	40.0 ± 6.0	0.002
Abdominal Circumference	47.6 ± 12.8	39.1 ± 13.5	0.03	29.3 ± 15.5	29.1 ± 16.2	0.9
